# Supplementary material for: Effect of BCG Revaccination on Occupationally Exposed Medical Personnel Vaccinated against SARS-CoV-2
Source: Cells. 2021 Nov 15;10(11):3179. doi: 10.3390/cells10113179 (PMC8617982; doi:10.3390/cells10113179)
Supplement: Supplementary file 1 [file cells-10-03179-s001.zip › cells-1407275-supplementary.pdf]

Supplementary Table S1. Comparison of serum cytokine concentrations 30 days after the application of the BCG vaccine or a placebo.

Cytokine	Placebo	BCG	p
IL-1β (pg/ml)	0.02(0.02-0.02)	0.135(0.02-0.021)	0.01
IL-2 (pg/ml)	4.035(3.18-7.1)	4.1(3.18-7.1)	0.83
IL-4 (pg/ml)	0.28(0.28-11.5)	0.28(0.28-2.55)	0.01
IL-5 (pg/ml)	0.36(0.21-0.53)	0.43(0.43-1.84)	0.45
IL-6 (pg/ml)	3.79(3.79-3.79)	3.79(3.79-3.79)	0.70
IL-12p70 (pg/ml)	0.0683(0.0683-0.0683)	0.135(0.02-0.21)	0.31
IL-13 (pg/ml)	1.76(1.76-1.76)	1.76(1.76-1.76)	0.54
IL-18 (pg/ml)	1.54(0.96-2.77)	3.065(0.34-7.52)	0.33
IFN-γ (pg/ml)	0.8211(0.8211-0.8211)	0.8211(0.8211-0.8211)	0.54
TNF-α (pg/ml)	0.34(0.1-1)	0.22(0.1-1)	0.76
GMCSF (pg/ml)	2.559(2.559-2.559)	2.559(2.559-2.559)	0.45

All values are expressed as medians (IQR).

Supplementary Table S2. Comparison of serum cytokine concentrations 30 days after the application of a placebo and 30 days after de second dose of SARS-Cov-2 vaccine

Cytokine	Placebo	Placebo+SARS-Cov-2 vaccine	p
IL-1β (pg/ml)	0.02(0.02-0.02)	0.36(0.21-0.538)	0.00001
IL-2 (pg/ml)	4.035(3.18-7.1)	7.1(7.1-7.1)	0.003
IL-4 (pg/ml)	0.28(0.28-11.5)	2.86(2.22-4.43)	0.4777
IL-5 (pg/ml)	0.36(0.21-0.53)	0.21(0.21-0.21)	0.0095
IL-6 (pg/ml)	3.79(3.79-3.79)	3.79(3.79-8.1)	0.091
IL-12p70 (pg/ml)	0.0683(0.0683-0.0683)	0.0683(0.0683-0.0683)	0.145
IL-13 (pg/ml)	1.76(1.76-1.76)	1.76(1.76-2.5)	0.360
IL-18 (pg/ml)	1.54(0.96-2.77)	8.0348(2.61-15.49)	0.0013
IFN-γ (pg/ml)	0.8211(0.8211-0.8211)	0.8211(0.8211-2.0327)	0.0017
TNF-α (pg/ml)	0.34(0.1-1)	1.944(0.1-3.67)	0.0153
GMCSF (pg/ml)	2.559(2.559-2.559)	2.559(2.559-12.6)	0.106

All values are expressed as medians (IQR).

Supplementary Table S3. Comparison of serum cytokine concentrations 30 days after the application of a BCG vaccine and 30 days after de second dose of SARS-Cov-2 vaccine

Cytokine	BCG	BCG+SARS-Cov-2 vaccine	P
IL-1β (pg/ml)	0.135(0.02-0.021)	0.538(0.472-0.672)	0.00001
IL-2 (pg/ml)	4.1(3.18-7.1)	7.1(7.1-7.1)	0.0001
IL-4 (pg/ml)	0.28(0.28-2.55)	11.5(11.5-11.5)	0.00001
IL-5 (pg/ml)	0.43(0.43-1.84)	1.74(0.93-3.68)	0.00001
IL-6 (pg/ml)	3.79(3.79-3.79)	8.1(8.1-8.1)	0.00001
IL-12p70 (pg/ml)	0.135(0.02-0.21)	0.5388(0.472-0.672)	0.00001
IL-13 (pg/ml)	1.76(1.76-1.76)	2.5(2.5-2.5)	0.00001
IL-18 (pg/ml)	3.065(0.34-7.52)	23(8.97-43.42)	0.00001
IFN-γ (pg/ml)	0.8211(0.8211-0.8211)	7.7216(3.272-12.3844)	0.00001
TNF-α (pg/ml)	0.22(0.1-1)	5.1777(1.245-6.3)	0.00001
GMCSF (pg/ml)	2.559(2.559-2.559)	12.6(12.6-12.6)	0.00001

All values are expressed as medians (IQR).
